# Percutaneous Vertebroplasty with Side‐Opening Cannula or Front‐Opening Cannula in the Treatment of Kummell Disease?

**DOI:** 10.1111/os.12730

**Published:** 2020-07-07

**Authors:** Xi‐fa Wu, Yong Ping, Xiang‐qin Zeng, Yong Feng, Zhen Wang, Tao Li, Dong‐jin Wu

**Affiliations:** ^1^ Department of Spinal Surgery Zibo Central Hospital ZiBo China; ^2^ Department of Orthopaedics Rizhao Central Hospital Rizhao China; ^3^ Department of Radiology Zibo Central Hospital ZiBo China; ^4^ Department of Orthopaedics Chongqing University Central Hospital Chongqing China; ^5^ Department of Spinal Surgery The Second Hospital of Shandong University Jinan China

**Keywords:** Complication, Kummell disease, Percutaneous vertebroplasty, Side‐opening cannula

## Abstract

**Objective:**

To explore the effect of bone cement distribution, cement leakage, and clinical outcomes with side‐opening cannula for bone cement injection in percutaneous vertebroplasty (PVP) in treatment of Kummell disease.

**Methods:**

A prospective study of patients with Kummell disease undergoing PVP was conducted from April 2012 to September 2017. In total, 43 patients (11 males, 32 females) with Kummell disease who received bilateral PVP were included in the study. The patients were divided into front‐opening cannulas (FOC) group with front‐opening cannulas and side‐opening cannulas (SOC) group with side‐opening cannulas. All patients were followed up for 6 months. The patient general information such as gender, age, bone density, compression ratio, operative time, and location of fracture vertebrae were recorded. Visual analogue scale (VAS), Oswestry Disability Index (ODI), bone cement distribution, radiation exposure time, bone cement leakage rate and vertebral height, and kyphosis angle were measured and compared for two groups before surgery, 1 day and 6 months after surgery.

**Results:**

A total of 43 patients were enrolled, including 11 males and 32 females, aged 61–84 years. The bone density (T value) was 2.5 ± 0.6 in FOC group and 2.4 ± 0.6 in SOC group (*P* > 0.05). The compression ratio and operative time were 36.1% ± 13.0%, 39.3 ± 7.9 min in FOC group and 35.2% ± 13.7%, 40.0 ± 10.7 min in SOC group (*P* > 0.05). There was no significance between FOC and SOC groups in the location of fracture vertebrae. All patients underwent at least 6 months of follow‐up. At 6 months postoperatively, the VAS and ODI were significantly higher in the FOC group (3.0 ± 0.8, 35.7% ± 2.1%) than in the SOC group (1.3 ± 0.4, 18.6% ± 2.4%) (*P* < 0.05). The cement leakage rate of the SOC group was 4.8%, which was lower than that of the FOC group (31.8%, *P* < 0.05), and the bone cement distribution ratio was higher than that of the FOC group (63.1% ± 7.9% *vs* 40.5% ± 8.6%, *P* < 0.05). At 6 months after operation, the height of the anterior and posterior vertebral bodies of the patients in the SOC group restored better than the FOC group (anterior SOC: FOC 5.1 ± 0.5 mm *vs* 4.5 ± 0.5 mm; posterior SOC: FOC 0.6 ± 0.1 mm *vs* 0.3 ± 0.1 mm, *P* < 0.05), and the kyphosis correction was more obvious than patients in FOC group (SOC: FOC 8.5° ± 1.4° *vs* 4.6° ± 0.8°, *P* < 0.05).

**Conclusion:**

Percutaneous vertebroplasty with side‐opening cannula is safe and effective in avoiding bone cement leakage, improving bone cement distribution, and restoring vertebral height.

## Introduction

The incidence of osteoporosis in the elderly increases with age^1^. The prevalence of osteoporosis in women was 15% in France, 16% in the USA, 9% in the United Kingdom, and 38% in Japan, while the prevalence was 8% in France, 1% in the United Kingdom, and 4% in Japan in men^2^. According to reports, the incidence of osteoporosis in China's population over 50 years of age accounts for about 34.65%, and the incidence of related fractures is about 1/3, which is more common in postmenopausal women^3^. Osteoporotic vertebral compression fracture (OVCF) occurs frequently in elderly patients with osteoporosis^4^. The low back pain with or without leg pain was the primary complaint of OVCF patients. OVCF can lead to fracture dislocation of the spine causing spinal instability and spinal cord damage^5^. OVCF affects patients' quality of life. Kummell disease refers to vertebral body collapse with a history of minor trauma and is caused by osteonecrosis of the vertebral body which is a special type of osteoporotic vertebral compression fracture and usually occurs in elderly patients with osteoporosis^6^. Since the first description by Dr. Hermann Kümmell in 1891, the main clinical manifestations of Kummell disease are persistent low back pain and kyphosis.

The conservative interventions for Kummell disease may lead to a higher rate of major complications such as increased osteoporosis, pneumonia, hemorrhoids, venous thrombosis, infection, and even lower limb paralysis^7^. Percutaneous vertebroplasty (PVP) is a minimally invasive surgical procedure and conventional treatment for patients with OVCFs^8^. PVP can partially or completely alleviate the back pain and improve activities of patients by the injection of polymethyl methacrylate (PMMA) bone cement into the fractured vertebra body via percutaneously cannula. This has been proven to be an effective minimally invasive surgical procedure for OVCF. However, the major concern arising from PVP is the leakage of bone cement. Bone cement leakage can also cause serious complications. If it leaks into the spinal canal, it can cause compression of the spinal cord and nerve roots, leading to severe pain or paraplegia of the lower extremities^9^. Leakage into the blood vessels can lead to pulmonary embolism. The incidence of PVP associated bone cement leakage ranges from 7%–82% as reported in the literature^10^. Although high‐viscosity bone cement can be used to reduce leakage, its operation time is too long and the injection pressure is too large, which is not conducive to surgical operation^11^. Thus, how to avoid the bone cement leakage is a major problem for surgeons in the procedure of PVP.

Because the opening side is located at the front end of the cannula in the traditional PVP operation procedure, the bone cement injected in the vertebral body is mostly limited to the anterior side. Therefore the center and the two sides of the vertebral body are less dispersed with bone cement, and the biomechanical function may not be restored. Uneven distribution of bone cement in the fractured vertebra body could cause the incomplete integration of bone cement cancellous bone^12^.

This untight combination of bone cement cancellous bone probably causes bone cement loosening, spinal instability, and persistent postoperative back pain. The insufficient cement distribution in the fractured area resulted in unrelieved pain after PVP in treatment of osteoporotic vertebral compression fractures^13^. Therefore, the uneven distribution of bone cement in the vertebral body is one of the important factors affecting the prognosis of PVP surgery. How to solve the uniform distribution of bone cement in the vertebral body is also a current clinical problem. In order to avoid the bone cement leakage and uneven distribution in the procedures of PVP, we used a modified side‐opening cannula (SOC) for cement injection in PVP. The successful application of SOC will provide a new bone cement injection method for PVP surgery, which may improve the success rate and prognosis of PVP surgery.

We hypothesized that PVP surgery using SOC would allow better bone cement distribution, avoid bone cement leakage, and improve the kyphosis and outcome compared to front‐opening cannulas (FOC) in patients with Kummell disease. Therefore, the purpose of this study was: (i) to assess the patients' gender, age, bone density, compression ratio, and location of fracture vertebrae, to ensure that patients were randomly assigned to the FOC and SOC groups, and the general information is comparable; (ii) to follow up and analyze the clinical outcomes of visual analogue scale (VAS), operation time, radiation exposure times, bone cement volume, Oswestry Disability Index (ODI), and cement leakage ratio between the FOC and SOC groups after PVP surgery; and (iii) to analyze radiographic outcomes of bone cement distribution, anterior vertebral height, posterior vertebral height, and improvement of Cobb angle between the FOC and SOC groups.

## Materials and Methods

### *Patients Selection, Inclusion and Exclusion Criteria*

This was a RCT study conducted between April 2012 and September 2017 at our hospital. This study was approved by the Medical Ethics Committee of Zibo Central Hospital, and complied with the World Medical Association Declaration of Helsinki on Ethical Principles for Medical Research Involving Humans.

Patients inclusion criteria were as follows: (i) the diagnoses of the patients were stage I or II Kummell disease with no nerve compression symptoms and single vertebral body lesion. Patients with focal back pain with or without the history of minor trauma, unresponsive to at least 6 weeks of conservative therapy. X‐ray examination showed significant wedge deformation of the vertebral body. The computed tomography (CT) scan showed the intravertebral vacuum cleft (IVC) phenomenon, intact posterior wall of the vertebral body and without pedicle fracture. Low signal on T1 and high signal on T2‐weighted MRI image were found; (ii) patients receive unilateral or bilateral PVP surgery; (iii) the parameters of bone density, compression ratio, location of fracture vertebrae in two groups before surgery were compared; (iv) VAS, operation time, radiation exposure times, bone cement volume, ODI, cement leakage ratio, bone cement distribution, vertebral height, and Cobb angle were measured; and (v) a RCT study.

Study exclusion criteria included: (i) pathologic fractures; (ii) primary and secondary bone tumors; (iii) bone tuberculosis; and (iv) patients with severe respiratory and circulatory diseases.

### *Study Design*

Between April 2012 and September 2017, 43 patients who were diagnosed with Kummell disease were recruited in this study. All the patients provided written informed consent before being included in the study. Forty‐three patients were randomly split into two groups based on the cannula used, with 22 cases in FOC group and 21 cases in SOC group. Twenty‐two patients received PVP using FOC in the FOC group while 21 patients in the SOC group received PVP surgery using SOC from April 2012 to September 2017. Demographic data and operative data were collected and shown in Table 1.

**Table 1:** Patient general information of the two groups

Groups	Gender (F/M)	Age (years)	Bone density (T‐score)	Compression ratio (%)	Location of fracture vertebrae (cases)		
					Thoracic	Thoracolumbar	Lumbar
FOC group (*n* = 22)	16/6	72.5 ± 8.7	2.5 ± 0.6	36.1 ± 13.0	5	12	5
SOC group (*n* = 21)	16/5	72.9 ± 11.1	2.4 ± 0.6	35.2 ± 13.7	4	13	4
*t*/*χ* ^2^	0.068	0.133	0.616	0.206	0.239		
*P* value	0.795	0.895	0.542	0.837	0.887		

### *Bone Cement Injection Devices*

**Figure 1:**
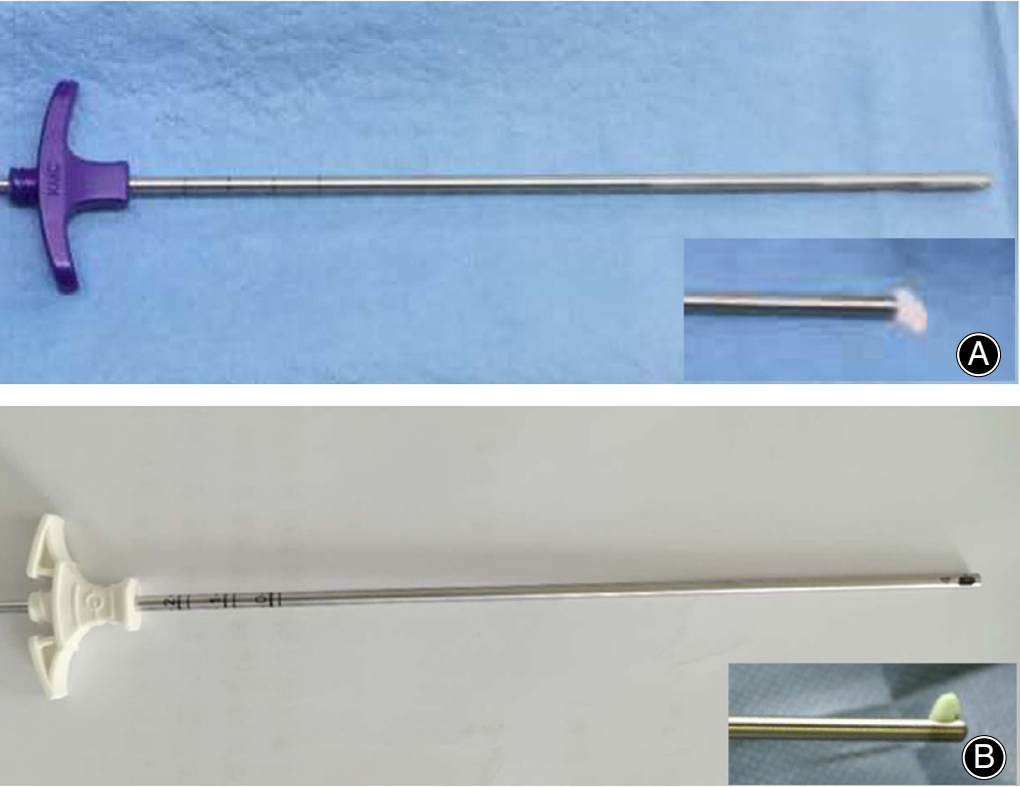
The injection cannulas in this study. (A) side‐opening cannula (B) front‐opening cannula

In order to solve the problem of the distribution of bone cement in the vertebral body after injection, we have adopted a modified side‐opening cannula (SOC) PVP injection device. As shown in Fig. 1, we can see an elliptical opening on the side of the distal end of the cannula. In the *in vitro* test, it can be seen that the bone cement flows out from the side and forms an angle of about 90° with the cannula. Through this side‐opening cannula, the surgeon rotates the cannula with the handle so that the bone cement can be evenly distributed in the cancellous bone and cavity in all directions. The traditional bone cement injection cannula has a front opening that bone cement flows out from the circular opening at the top end of the cannula, and the outflow direction of the bone cement forms an angle of 180° with the cannula. The FOC and SOC cannulas and their accessorial devices are commonly used in PVP.

### *Surgical Procedures*

#### *Anesthesia and Surgical Position*

The patients were placed in a prone position. All procedures were performed on using a C‐arm angiographic unit. After conventional skin disinfection, 1% lidocaine was injected from the puncture site to the periosteum for local anesthesia.

#### *Approach and Exposure*

Puncture point location and angle for pedicle were determined from the CT scan image. The puncher needle was inserted into the pedicle at 10 o'clock in the right and 2 o'clock in the left. The needle was gradually inserted into the vertebral body from the cortex and pedicle at an angle of 10°–15°. Ensure that the puncture needle was completely located in the bony channel under X‐ray. The needle top was located 5 mm behind the front edge of the vertebra. Then, the puncture needle was removed and a working cannula was constructed.

#### *Bone Cement Injection*

After the bone cement was mixed and solidified to the drawing state, the FOC and SOC cannula were used for cement injection. The procedure was monitored under fluoroscopy in digital subtraction angiography (DSA). When using the SOC cannula, the opening side is toward the opposite side of the vertebral body. The direction of bone cement dispersion was closely observed during surgery. When the bone cement reaches the edge of the vertebral body, the sleeve was rotated in time to adjust the direction of the opening. When the FOC cannula was used for injection of bone cement, the cannula was gradually pulled out according to the dispersion degree of the bone cement. After the bone cement was completely solidified, the working channel was removed. Both groups of patients used the same viscosity and brand of bone cement.

### *Data Collection and Outcome Evaluation*

The raw observation data for the two groups were recorded on the day before surgery and 1 day and 6 months after surgery, and were compared as follows.

#### *Operative Time*

The time of surgery was recorded from the beginning of puncture until surgical closure, which could reflect the effect of two types of cannula on the operation time.

#### *Visual Analogue Scale (VAS) Scores*

The VAS is the most commonly used questionnaire for quantification of pain. It is a continuous scale comprised of a horizontal or vertical line, usually 10 cm in length. For pain intensity, the scale is most commonly anchored by “no pain” (score of 0) and “pain as bad as it could be” (score of 10). A score of 0 is considered as no pain, 1–3 is mild pain, 4–6 is moderate pain, and 7–10 is severe pain.

#### *Vertebral Cobb Angle Improvement*

The Cobb angle is a measure of the curvature of the spine in degrees. Which measured from the superior endplate of a superior vertebra to the inferior endplate of an inferior vertebra at a particular region of the vertebral column. The vertebral Cobb angle improvement was calculated by preoperative Cobb angle ‐ postoperative Cobb angle. The better the Cobb angle improvement, the better the kyphosis correction.

#### *Oswestry Disability Index (ODI)*

Oswestry Disability Index (ODI) is a principal condition‐specific outcome measure used in the management of spinal disorders, and to assess patient progress in routine clinical practice. The ODI score system includes 10 sections: pain intensity, personal care, lifting, walking, sitting, standing, sleeping, sex life, social life, and traveling. For each section of six statements the total score is 5. Intervening statements are scored according to rank. If more than one box is marked in each section, take the highest score. If all 10 sections are completed the score is calculated as follows: total scored out of total possible score × 100. If one section is missed (or not applicable) the score is calculated: (total score/[5 × number of questions answered]) × 100%. A score of 0%–20% is considered mild dysfunction, 21%–40% is moderate dysfunction, 41%–60% is severe dysfunction, and 61%–80% is considered as disability. For cases with score of 81%–100%, the patient is either long‐term bedridden or exaggerating the impact of pain on their life.

#### *Bone Cement Leakage*

Bone cement leakage refers to the situation where bone cement flows from the vertebral body to the outside of the vertebral body. Bone cement leakage can also lead to serious complications after PVP. This is also an index to evaluate the effect of PVP surgery. Postoperative X‐ray film was used for cement leakage evaluation according to the criteria proposed by Yeom *et al*.^14^. Cement leakage B type: bone cement leaks along the vertebral basilar to the posterior edge of the vertebral body. Cement leakage C type: bone cement leaks along the endplate cortical defect to the intervertebral space and the outside of the vertebral body. Cement leakage S type: bone cement leaks along the intervertebral vein to the periphery of the vertebral body.

#### *Restoration Rate of Vertebral Height*

**Figure 2:**
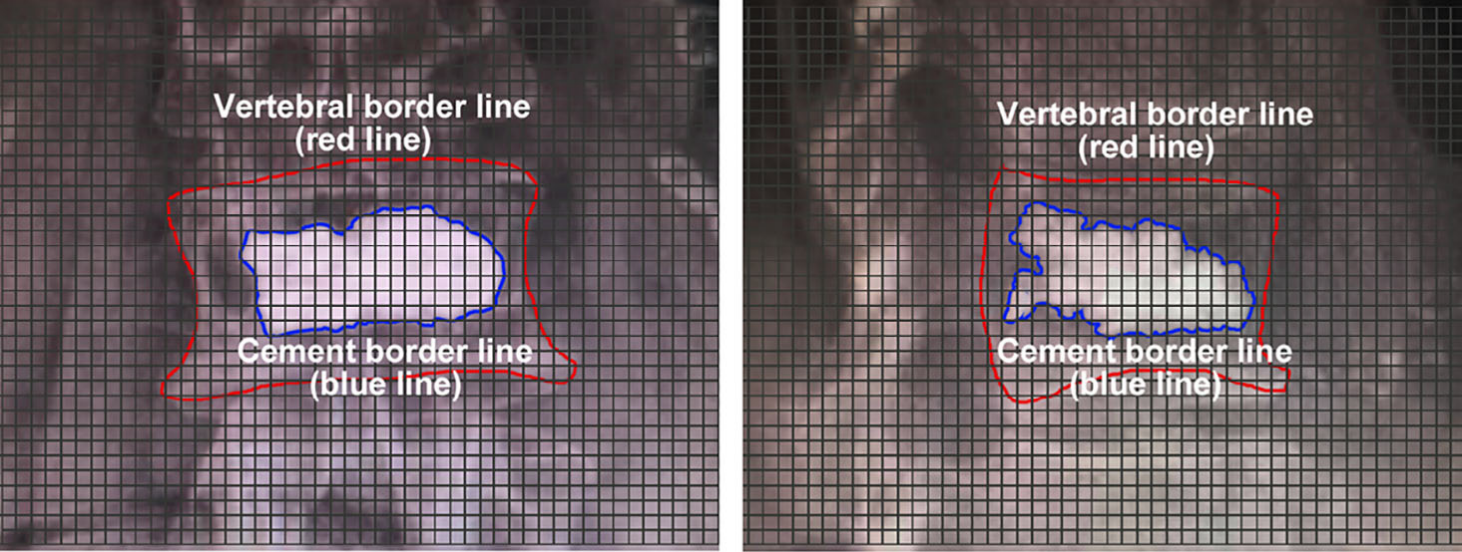
Measurement of cement distribution. According to the anteroposterior and lateral radiographs of the affected spine, the edges of the vertebral bodies are outlined with a red line, and the edges of the bone cement are outlined with a blue line. The entire plane is divided into squares, and the distribution of the bone cement is calculated according to the number of squares occupied by the blue and red lines.

Vertebral height restoration correlates with improvements in pain, disability, and quality of life in patients with Kummell disease. The restoration rate of vertebral height = (postoperative vertebral height ‐ preoperative vertebral height) / (predicted primary vertebral height ‐ preoperative vertebral height) × 100% (Fig. 2).

#### *Cement Distribution*

**Figure 3:**
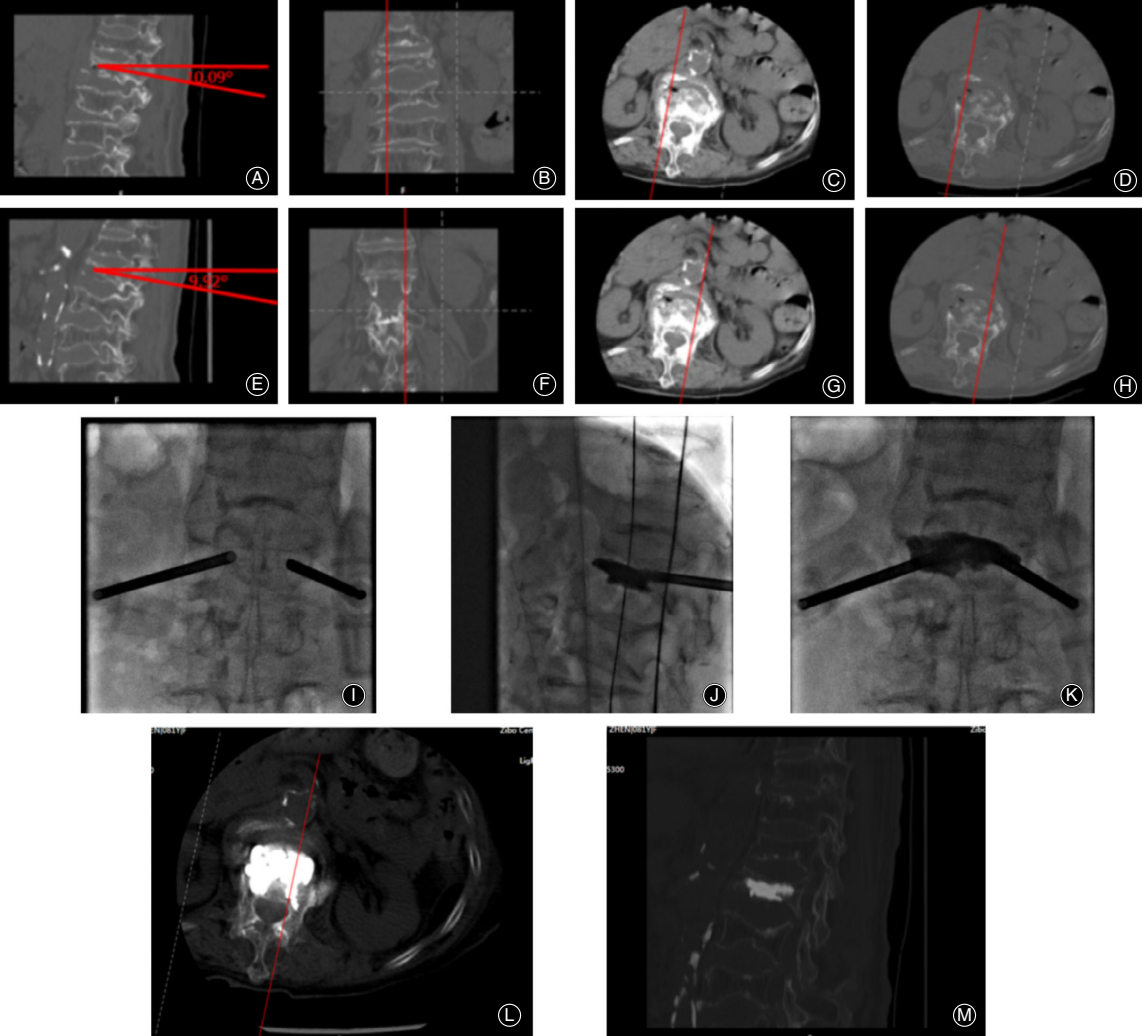
PVP surgical procedure with side‐opening cannula. (A–D) The angle and position of the right side of the vertebral body are determined based on the CT scan results. (E–H) The angle and position of the left side of the vertebral body are determined based on the CT scan results. (I) An anteroposterior X‐ray film after implantation of the cannulas on both sides. (J–K) The bone cements were gradually injected into both sides, and the cannulas were rotated to adjust the direction of the bone cement outlet to ensure uniform distribution in the vertebral body. The anteroposterior and lateral X‐ray film after bone cement injection. (L‐M) CT scan results showed that the bone cement was evenly distributed without leakage.

The cement distribution of the two groups was referred to as the mean ratio of bone cement areas to the whole vertebra area both in the lateral and anteroposterior views under X‐ray (Fig. 3). The X‐ray pictures were divided by a number of the same sized squares. The area of each square was 0.25 mm^2^. Different brightness areas were selected by the software automatically. The red line meant vertebral border and the blue line meant cement border. If the selected area was more than half the whole square, the area was calculated as 1. Otherwise, it was 0. Then the ratio of cement area to vertebral cross‐sectional area was estimated.

### *Statistical Analysis*

All data were collected by an independent observer and presented as mean ± standard deviation. The data were analyzed by SPSS v19.0 (SPSS™ Inc., Chicago, Illinois). Baseline demographic and operative data between the SOC and FOC groups were assessed by the Student *t*‐test and chi‐square test. Preoperative and postoperative VAS, ODI, vertebral Cobb angle, and cement leakage were compared by the *t*‐test. The statistical significance was defined as *P* < 0.05.

## Result

### *General Patient Information*

The baseline characteristics of both groups were similar (Table 1). In this study, there were 43 patients with Kummell disease who underwent PVP treatment and were followed up for at least 6 months. There are 11 males and 32 females. It can be seen from the gender distribution that the incidence of women's Kummell disease is higher than that of men. The FOC and SOC groups were randomly included in 22 patients and 21 patients, respectively. The mean ages of the FOC and SOC groups were 72.5 ± 8.7 years and 72.9 ± 11.1 years (*P* = 0.895). There were no significant differences in the compression ratio, gender, bone density, and location of fractured vertebrae between the two groups (*P* = 0.837 for compression ratio, *P* = 0.795 for gender, *P* = 0.542 for bone density, *P* = 0.794 for location of fractured vertebrae). The thoracolumbar segment is the major lesion location in the two groups.

### *Clinical Outcomes*

#### *VAS*

Both groups of patients successfully completed the PVP surgery. There were no serious complications. The preoperative pain was more obvious and the average VAS score reached 7.6 in both groups of patients. After PVP, the VAS scores of the two groups were significantly decreased at 1 day after surgery, average VAS scores were 2.0 and 1.9, respectively (*P* = 0.456). However, at the 6‐month follow‐up, the VAS score of the FOC group increased, while the VAS score of the SOC group decreased to 1.3 (*P* = 0.001).

#### *Operation and Radiation Exposure Times*

There were no significant differences in the operation time and radiation exposure time between the two groups (*P* = 0.605 for operation time and *P* = 0.511 for radiation exposure time).

#### *ODI*

The ODI score can be used to assess the degree of spinal dysfunction in patients. There was no significant difference in ODI scores between the two groups before surgery (FOC:SOC 78.3 ± 8.2 *vs* 78.4 ± 8.5, *P* = 0.925), but the ODI scores of the SOC group were significantly lower than those of the FOC group at 1 day and 6 months after surgery (FOC:SOC 36.5 ± 5.0 *vs* 29.9 ± 4.5, *P* = 0.001 for 1 day; 35.7 ± 2.1 *vs* 18.6 ± 2.4, *P* = 0.000 for 6 months).

#### *Cement Volume and Leakage Ratio*

In addition, the amount of injected bone cement in the SOC group was (4.8 ± 0.7) mL, which was higher than that of the FOC group (4.2 ± 0.9 mL) (*P* = 0.016). And the cement leakage rate was 4.8% in SOC group, which was lower than 31.8% of the FOC group (*P* = 0.023) (Table 2).

**Table 2:** The comparison of clinical outcomes between two groups

Groups	VAS			Operation time (min)	Radiation exposure times (s)	Bone cement volume (mL)	ODI index (%)			Cement leakage ratio (cases [%])
	Preoperation	1 Day post‐operation	6 Months post‐operation				Preoperation	1 Day post‐operation	6 Months post‐operation	
FOC group (*n* = 22)	7.6 ± 1.3	2.0 ± 0.8	3.0 ± 0.8	32.2 ± 5.9	14.8 ± 2.4	4.2 ± 0.9	78.3 ± 8.2	36.5 ± 5.0	35.7 ± 2.1	7 (31.8)
SOC group (*n* = 21)	7.6 ± 1.5	1.9 ± 0.9	1.3 ± 0.4	31.0 ± 6.6	15.0 ± 2.5	4.8 ± 0.7	78.4 ± 8.5	29.9 ± 4.5	18.6 ± 2.4	1(4.8)
*t*/*χ* ^2^	0.057	0.749	7.918	0.519	0.663	2.525	0.094	4.524	21.502	5.194
*P* value	0.955	0.456	0.001	0.605	0.511	0.016	0.925	0.001	0.000	0.023

### *Radiographic Outcomes*

#### *Cement Distribution*

According to the X‐ray test at 1 day post‐operation, we used the grid division method to analyze the bone cement distribution of the two groups. Based on the results of the X‐ray, we calculated that the bone cement distribution ratio of patients in the SOC group was 63.1%, which was significantly higher than the 40.5% in the FOC group (*P* = 0.000).

#### *Vertebral Height and Restoration*

There was no significant difference between the two groups in the anterior and posterior height of the vertebral body before the operation according to the lateral X‐ray film (*P* = 0.719 for anterior and *P* = 0.934 for posterior). After 6 months of follow‐up, the anterior vertebral height of the SOC group restored (5.1 ± 0.5) mm, and was significantly higher than that of FOC group (4.5 ± 0.5 mm) (*P* = 0.001). There was statistical difference in the recovery of posterior vertebral height between the two groups (FOC:SOC, 0.3 ± 0.1 *vs* 0.6 ± 0.1, *P* = 0.000).

#### *Cobb Angle Improvement*

The Cobb angle restoration of the vertebral body in the SOC group was also significantly higher than that in the FOC group (FOC:SOC, 4.6º ± 0.8º *vs* 8.5º ± 1.4º, *P* = 0.000) (Table 3). The above results showed that side‐opening cannula injection can effectively promote bone cement distribution and correction of kyphosis.

**Table 3:** The comparison of radiographic outcomes between two groups (mean ± SD)

Groups	Bone cement distribution (%)	Preoperative anterior vertebralheight (mm)	Preoperative posterior vertebralheight (mm)	Anterior vertebralheight restoration (mm)	Posteriorvertebralheight restoration (mm)	Improvement of Cobb angle (°)
FOC group (*n* = 22)	40.5 ± 8.6	13.0 ± 1.4	20.9 ± 1.5	4.5 ± 0.5	0.3 ± 0.1	4.6 ± 0.8
SOC group (*n* = 21)	63.1 ± 7.9	12.9 ± 1.1	20.8 ± 1.5	5.1 ± 0.5	0.6 ± 0.1	8.5 ± 1.4
*t* value	8.992	0.363	0.083	3.589	11.883	11.604
*P* value	0.000	0.719	0.934	0.001	0.000	0.000

### *Complications*

There were seven cases in the FOC group and one case in the SOC group with cement leakage. These patients have less degree of bone cement leakage and the site of leakage was intervertebral disc. There were no clinical manifestations in these patients. One patient in the FOC group had adjacent vertebral fracture in L_2_ level, the low back pain disappeared after bed rest and oral non‐steroidal anti‐inflammatory drugs.

## Discussion

### *Epidemiology and Characteristics of Kummell Disease*

Intractable low back pain is the main clinical manifestation of Kummell disease, and there may be neurological symptoms in severe cases^15^. The incidence rates of Kummell disease in elderly OVCF cases are 7%–37%^7^. The Kummell disease occurs mostly in the thoracolumbar vertebral body, which may be related to the following factors. The first factor is stress changes in the fracture vertebral body. The vertebral body kyphosis angle increases progressively after osteoporotic compression fracture. The kyphosis deforms makes the center of gravity of the spine move forward and changes the biomechanical environment of the vertebral body^16^. This abnormal stress distribution further aggravates the compression. In addition, the thoracolumbar region is the transitional zone of the physiological curvature of the spine. The abnormal dynamic load and the micro‐motion‐induced fracture healing of the fracture are delayed, and even eventually occur fracture nonunion, pseudo articular formation, and vertebral body collapse. The second factor is the fracture vertebral ischemic necrosis. Osteoporotic vertebral fractures occur mostly in the anterior column of the vertebral body, destroying the normal nutritional blood supply of the vertebral body, resulting in avascular necrosis of the trabeculae, leading to the absorption of the trabecular bone in the central region of the necrosis, forming an “intravertebral vacuum cleft”^17^. This causes further fracture of the vertebral body after minor trauma, forming a malignant circulation of ischemic‐fracture^18^. Among the patients included in this study, the main region of Kummell disease was also in the thoracolumbar region of the spine.

### *Clinical Research Progress of PVP in Treatment of OVCF and Clinical Outcomes of PVP using SOC in Kummell Disease*

The conservative treatment of Kummell disease is the same as OVCF, including bed rest, drug symptomatic treatment, and wearing a brace^19^. However conservative treatment is usually not effective in relieving symptoms. PVP and PKP are highly effective in relieving pain and improve the prognosis of OVCF^20^, ^21^. They have advantages including vertebral body formation, minimally invasive, rapid pain relief, quick recovery, and have been widely used in the treatment of OVCF. PKP can form a space in fractured vertebra by balloon dilatation, and the injected bone cement is easy to form a mass. The bone cement is poorly distributed in the surrounding cancellous bone, and the bone cement ball in the center of the dilated space is easy to cause loosening and vertebral body fracture again. PVP also has a high rate of cement leakage rate in the treatment of OVCF or Kummell disease. Bone cement leakage is one of the main factors affecting postoperative complications of PVP.

Han *et al*. pointed out that patients with intravertebral vacuum cleft can experience 75% of cement leakage after PVP^22^. It was reported that bone cement leakage rate was 66.7% in PVP by analyzing patients with intravertebral vacuum cleft^23^. Compared with general osteoporotic fractures, Kummell disease has a high risk of bone cement leakage, long‐term bone cement loosening, and low back pain. Peh *et al*. reported that PVP treatment of Kummell disease results in a bone cement leakage rate up to 79%, and the cement leakage is related to vertebral body rupture^24^.

### *Significance of Intravertebral Vacuum Cleft Sign in Kummell Disease*

Many intravertebral vacuum clefts are directly connected to the adjacent intervertebral disc or at the anterior edge of the vertebral body, resulting in incomplete anterior cortical bone of the vertebral body. Bone cement leakage is mostly in the front of the intervertebral disc and vertebral body of Kummell disease. Cement leakage in the intervertebral disc is usually asymptomatic, but may increase the risk of secondary fractures in adjacent segmental vertebral bodies. Pulmonary embolism is the severe complication of bone cement leakage. Kim *et al*. pointed out that the incidence of pulmonary embolism in patients with intravertebral vacuum cleft sign was significantly lower than that in patients without intravertebral vacuum cleft^25^. The intravertebral vacuum cleft sign was considered as a protective factor for cement embolism events.

In the present study, we used two types of cannulas in PVP procedures, and the VAS scores of the patients in two groups were significantly improved after surgery. Also, there was no difference in VAS scores 1 day after surgery between groups. At 6 months after surgery, the VAS score of the patients in FOC group was higher than that of the SOC group, which may be related to the poor distribution of bone cement, the loosening of the bone cement mass in the fracture cleft, and the re‐fracture caused by vertebral instability. According to the ODI score of our study, we could also find that the SOC group had better spine function than the FOC group, which was consistent with the VAS score.

### *Avoid Cement Leakage*

How to reduce the high leakage rate of bone cement in PVP is still a hot issue in clinical work. Cement leakage in PVP treatment of Kummell disease is affected by the vertebral vacuum crevice, bone cement injection time, injection volume, and operation technology^26^. Bone cement injection site is one of the issues in PVP. Intravertebral vacuum cleft is the main source of pain in patients with Kummell disease, and it is also a risk factor for increasing bone cement leakage. The most important role of bone cement in vertebroplasty is to integrate the fractured vertebra body. The prognosis of PVP is closely related to the distribution of bone cement in the vertebral body^27^. Therefore, carefully assessed the size of the vertebral body, fracture line, and fracture degree, and planning puncture angle and depth by CT image, were important for PVP.

### *Radiographic Outcomes*

The application of the controlled opening side cannula in bone cement injection will achieve satisfied bone cement distribution. In this study, we used the one side puncture to reach the normal bone through the cavity area, and another side puncture to the cavity area, so that the bone cement that filled in the cavity area can be anchored with the bone cement in the normal cancellous bone. This combination of bone cement can avoid bone cement loosening and improve vertebral stability. Our results also showed that the distribution of bone cement in the SOC group was as high as 63.1%, which was significantly higher than 40.5% in the FOC group. At the same time, the degree of restoration of vertebral height and the degree of kyphosis correction in the SOC group were also better than the FOC group. Based on the particularity of the intravertebral vacuum cleft, the bone cement injection time in this study was set to the later stage of the bone cement drawing period. The bone cement in this period can not only be fully dispersed in the vertebral body, but is also not easy to leak into the paravertebral and blood vessels.

This method of injection made our bone cement leakage rate lower in this study. The amount of bone cement injection is not proportional to the degree of postoperative pain relief, and the amount of bone cement injection is an independent factor to increase the leakage complications. Excessive injection can increase the risk of re‐fracture in adjacent segments. The optimal amount of bone cement should fill the intravertebral vacuum cleft and disperse to the anterior 2/3 of the vertebral body^9^. In this study, we used a side‐opening cannula to avoid the crack of the fractured vertebra body, and the rotary‐side‐opening cannula could adjust direction of bone cement dispersion and reduce the leakage of bone cement. By adjusting the depth of the puncture cannula, we applied a phased injection method. That is, the targeted puncture cannula reached the fracture area, rotates the lateral opening direction, pushed a small amount of the drawing period bone cement, and filled the fracture gap. At intervals of 2 to 3 min, the bone cement was re‐perfused along the rear of the first injection site, and the bone cement putter was retracted 1 to 2 mm, and then injected until the bone cement was well dispersed.

### *Limitations*

We find several limitations in this study. The number of patients in this study was relatively small. Longer follow‐up is needed to evaluate the height loss of vertebral body in the long‐term and the correction effect of kyphosis after PVP using SOC.

### *Conclusion*

Both FOC and SOC can effectively improve short‐term low back pain in patients with Kummell disease in PVP. The SOC is also safe, effective, and easy to operate in PVP procedures. Compared with FOC, SOC can more effectively improve the distribution of bone cement in the vertebral body, restore the height of the vertebral body, and improve kyphosis without adding additional medical expenses. Meanwhile, SOC also has obvious advantages in avoiding bone cement leakage. In conclusion, SOC is a good choice for treating Kummell disease in PVP.

## Ethics Approval

All procedures performed in studies involving human participants were in accordance with the ethical standards of the institutional research committee and with the 1964 Declaration of Helsinki and its later amendments or comparable ethical standards.

## References

[1] Vandenbroucke A , Luyten FP , Flamaing J , Gielen E . Pharmacological treatment of osteoporosis in the oldest old. Clin Interv Aging, 2017, 12: 1065–1077. 28740372 10.2147/CIA.S131023 PMC5505539

[2] Wade SW , Strader C , Fitzpatrick LA , Anthony MS , O'Malley CD . Estimating prevalence of osteoporosis: examples from industrialized countries. Arch Osteoporos, 2014, 9: 182. 24847682 10.1007/s11657-014-0182-3

[3] Chen P , Li Z , Hu Y . Prevalence of osteoporosis in China: a meta‐analysis and systematic review. BMC Public Health, 2016, 16: 1039. 27716144 10.1186/s12889-016-3712-7 PMC5048652

[4] Nakamae T , Fujimoto Y , Yamada K , Hashimoto T , Olmarker K . Efficacy of percutaneous Vertebroplasty in the treatment of osteoporotic vertebral compression fractures with Intravertebral cleft. Open Orthop J, 2015, 9: 107–113. 26157525 10.2174/1874325001509010107 PMC4484235

[5] Park JH , Kang KC , Shin DE , Koh YG , Son JS , Kim BH . Preventive effects of conservative treatment with short‐term teriparatide on the progression of vertebral body collapse after osteoporotic vertebral compression fracture. Osteoporos Int, 2014, 25: 613–618. 23943161 10.1007/s00198-013-2458-7

[6] Kim HS , Adsul N , Bang JS , Singh R , Park CH , Jang IT . Refracture of Kummell disease combined with huge epidural hematoma after minor trauma. World Neurosurg, 2018, 120: 500–505. 30266694 10.1016/j.wneu.2018.09.130

[7] Liu F , Chen Z , Lou C , *et al* Anterior reconstruction versus posterior osteotomy in treating Kummell's disease with neurological deficits: a systematic review. Acta Orthop Traumatol Turc, 2018, 52: 283–288. 29803679 10.1016/j.aott.2018.05.002 PMC6146012

[8] Svedbom A , Alvares L , Cooper C , Marsh D , Strom O . Balloon kyphoplasty compared to vertebroplasty and nonsurgical management in patients hospitalised with acute osteoporotic vertebral compression fracture: a UK cost‐effectiveness analysis. Osteoporos Int, 2013, 24: 355–367. 22890362 10.1007/s00198-012-2102-y PMC3691631

[9] Hong SJ , Lee S , Yoon JS , Kim JH , Park YK . Analysis of intradiscal cement leakage during percutaneous vertebroplasty: multivariate study of risk factors emphasizing preoperative MR findings. J Neuroradiol, 2014, 41: 195–201. 24080117 10.1016/j.neurad.2013.07.004

[10] Martin DJ , Rad AE , Kallmes DF . Prevalence of extravertebral cement leakage after vertebroplasty: procedural documentation versus CT detection. Acta Radiol, 2012, 53: 569–572. 22637642 10.1258/ar.2012.120222

[11] Tang S , Fu W , Zhang H , Liang B . Efficacy and safety of high‐viscosity bone cement Vertebroplasty in treatment of osteoporotic vertebral compression fractures with Intravertebral cleft. World Neurosurg, 2019, 132: e739–e745. 31415893 10.1016/j.wneu.2019.08.029

[12] Kim DJ , Kim TW , Park KH , Chi MP , Kim JO . The proper volume and distribution of cement augmentation on percutaneous vertebroplasty. J Korean Neurosurg Soc, 2010, 48: 125–128. 20856660 10.3340/jkns.2010.48.2.125 PMC2941854

[13] Liang D , Ye LQ , Jiang XB , *et al* Biomechanical effects of cement distribution in the fractured area on osteoporotic vertebral compression fractures: a three‐dimensional finite element analysis. J Surg Res, 2015, 195: 246–256. 25634828 10.1016/j.jss.2014.12.053

[14] Yeom JS , Kim WJ , Choy WS , Lee CK , Chang BS , Kang JW . Leakage of cement in percutaneous transpedicular vertebroplasty for painful osteoporotic compression fractures. J Bone Joint Surg Br, 2003, 85: 83–89. 12585583 10.1302/0301-620x.85b1.13026

[15] Ma R , Chow R , Shen FH . Kummell's disease: delayed post‐traumatic osteonecrosis of the vertebral body. Eur Spine J, 2010, 19: 1065–1070. 19949820 10.1007/s00586-009-1205-4 PMC2900014

[16] Wang H , Ding W . Posterior vertebral column resection through unilateral osteotomy approach for old lumbar fracture combined with Kummell disease. World Neurosurg, 2018, 109: 147–151. 28974411 10.1016/j.wneu.2017.09.148

[17] He D , Yu W , Chen Z , Li L , Zhu K , Fan S . Pathogenesis of the intravertebral vacuum of Kummell's disease. Exp Ther Med, 2016, 12: 879–882. 27446290 10.3892/etm.2016.3369 PMC4950591

[18] Matzaroglou C , Georgiou CS , Assimakopoulos K , *et al* Kummell's disease: pathophysiology, diagnosis, treatment and the role of nuclear medicine. Rationale according to our experience. Hell J Nucl Med, 2011, 14: 291–299. 22087452

[19] Kato T , Inose H , Ichimura S , *et al* Comparison of rigid and soft‐brace treatments for acute osteoporotic vertebral compression fracture: a prospective, randomized, multicenter study. J Clin Med, 2019, 8 pii: E198. 10.3390/jcm8020198 PMC6406237 30736328

[20] Zhu J , Zhang K , Luo K , *et al* Mineralized collagen modified Polymethyl methacrylate bone cement for osteoporotic compression vertebral fracture at 1‐year follow‐up. Spine (Phila Pa 1976), 2019, 44: 827–838. 30601358 10.1097/BRS.0000000000002971

[21] Youn MS , Shin JK , Goh TS , Lee JS . Minimally invasive percutaneous endoscopic treatment for acute pyogenic spondylodiscitis following vertebroplasty. Eur Spine J, 2018, 27: 458–464. 10.1007/s00586-018-5478-3 29356984

[22] Han S , Park HS , Pee YH , Oh SH , Jang IT . The clinical characteristics of lower lumbar osteoporotic compression fractures treated by percutaneous vertebroplasty: a comparative analysis of 120 cases. Korean J Spine, 2013, 10: 221–226. 24891852 10.14245/kjs.2013.10.4.221 PMC4040645

[23] Wang H , Sribastav SS , Ye F , *et al* Comparison of percutaneous Vertebroplasty and balloon Kyphoplasty for the treatment of single level vertebral compression fractures: a meta‐analysis of the literature. Pain Physician, 2015, 18: 209–222. 26000665

[24] Peh WC , Gilula LA . Percutaneous vertebroplasty: indications, contraindications, and technique. Br J Radiol, 2003, 76: 69–75. 12595329 10.1259/bjr/10254271

[25] Kim MH , Lee AS , Min SH , Yoon SH . Risk factors of new compression fractures in adjacent vertebrae after percutaneous vertebroplasty. Asian Spine J, 2011, 5: 180–187. 21892391 10.4184/asj.2011.5.3.180 PMC3159067

[26] Park JW , Park JH , Jeon HJ , Lee JY , Cho BM , Park SH . Kummell's disease treated with percutaneous Vertebroplasty: minimum 1 year follow‐up. Korean J Neurotrauma, 2017, 13: 119–123. 29201845 10.13004/kjnt.2017.13.2.119 PMC5702746

[27] Tanigawa N , Komemushi A , Kariya S , *et al* Relationship between cement distribution pattern and new compression fracture after percutaneous vertebroplasty. AJR Am J Roentgenol, 2007, 189: W348–W352. 18029848 10.2214/AJR.07.2186

